# Development of Novel Composite Membranes in Water/Wastewater Treatment

**DOI:** 10.3390/membranes12030260

**Published:** 2022-02-24

**Authors:** Hazim Qiblawey

**Affiliations:** Department of Chemical Engineering, College of Engineering, Qatar University, Doha P.O. Box 2713, Qatar; hazim@qu.edu.qa

Composite membranes have attracted significant attention due to their flexibility in having more than one layer, with many materials being used to form the membrane. This will enable the scientific and the industrial community to have produce tailored membranes for specific purposes. This flexibility in composite membrane synthesis has allowed them to be used in a wide scope of separation and beneficiation processes for both liquid and gaseous applications. Current commercial membranes such as those for nanofiltration and reverse osmosis are predominantly thin composite materials [1,2].

This Special Issue titled “Development of Novel Composite Membranes in Water/Wastewater Treatment” in the journal *Membranes* aims to present the state-of-the-art contributions from authors producing novel composite membranes from synthesis to applications in water and wastewater treatment to assess recent developments in sustainable and environmentally friendly approaches. 

Advanced materials such as Graphene oxide (GO) have been widely used as a filler in membrane applications due to their high oxygen content. This will improve the membrane’s antifouling properties and its functionality including permeability and selectivity. The modified Hummer’s method is the one usually used to produce GO [3,4]. Two studies were considered in this Special Issue. The first one is by Alkhouzaam et al. [5] who produced Graphene oxide nanoparticles using the modified Hummer’s method. The obtained GO NP were amine functionalized using the bio-inspired polydopamine (PDA) to increase their hydrophilicity, dispersibility in aqueous and organic solvents, antibacterial properties, surface area, adsorption capacity, mechanical and thermal stability [6,7]. Both NPs were investigated using different types of characterization techniques to provide comprehensive analysis. The structural change was evaluated using XRD, FTIR-UATR, Raman spectroscopy, SEM and TEM. The XRD patterns of pristine GO and GO-PDA NPs showed incomplete oxidation of graphite to GO, which is one of the drawbacks of Hummers-based methods. Different diffraction peaks proved the successful functionalization of GO with PDA. SEM and TEM images showed two distinct morphologies of GO and GO-PDA NPs, and created a successful grafting of PDA on the GO surface. The FTIR spectra of the pristine GO confirm the oxidation of graphite owing to the presence of several bands attributed to oxygen functionalization. Emergence of new amide bands confirmed successful functionalization of GO NPs. Raman spectra of the synthesized GO and GO-PDA showed the crystallite size to be 10.9 and 15.2 nm for the pristine GO and GO-PDA, respectively. Further analysis of the Raman spectra provided quantitative and qualitative information about the properties of GO such as details of defects and the number of layers. Surface elemental compositions were explored using XPS survey spectra and showed that GO was successfully aminated with PDA. Dispersibility photographs of GO and GO-PDA suspensions after sonication were presented. While pristine GO exhibited good dispersibility in some solvents, GO-PDA showed high dispersion in all solvents. This high dispersibility of GO-PDA in polar and nonpolar solvents is a plus value for various applications over the pristine GO. The pristine GO and GO-PDA were deposited on a PS-30 substrate using pressurized assisted self-assembly (PAS) to investigate its hydrophilicity. Finally, the contact angle was much lowered to 27.8° (compared to 76.7° for PS), with the assembly of the GO-PDA layer suggesting higher hydrophilicity of GO-PDA than the pristine GO NPs. The authors come to a final conclusion that GO-PDA has potential as nanofiller for modifying different membrane materials and types due to its higher hydrophilicity and dispersibility [8].

The second study by Kadhim et al. [9] used graphene oxide nanoparticles (GO-NPs) to modify the polyethersulfone (PES) membrane to remove two types of dyes (Acid Black and Rose Bengal). Seven membranes were prepared with GO ranging from 0 to 2%. The performance and antifouling properties of the membranes were studied using FTIR, SEM, AFM, water permeation flux and dye removal. SEM cross-sectional images show that all PES membranes have typically symmetrical and porous structures. Increasing the GO content resulted in the formation of larger pores due to the high hydrophilicity of GO, which is expected to enhance the water flux. The FTIR analysis confirmed the formation of the high oxygen bonding within the membrane matrix. This improved the membranes hydrophilicity from 60.82° for the PES membrane to the highest water contact angle of 39.21° after adding 0.5% GO. The AFM images showed no clear trend of the effect of GO on membrane roughness. However, the mean pore radius of the membrane was highly enhanced with increasing GO content. Addition of GO to the membrane matrix proved to increase water flux and to improve the dye rejection significantly compared to the pristine PES membranes. The collective tests showed that the membrane with 0.5% GO has the best performance; therefore, the authors further investigated the effect the solution pH on the membrane rejection and stability of the membrane after long periods of operations reaching 26 days.

Vijitha et al. [10] established a simple method for the fabrication of polyelectrolyte membranes (PEMs) with sulfonate functionalized pectin and poly(vinyl alcohol)(PVA). Two membranes (PPCAM and PPCSB) were synthesized by a blending PVA solution and a solution of graft-copolymers (2 g of PC-g-AMPS/PC-g-SVBS). The blended solutions were poured onto a clean glass plate to form the membranes. The membranes were then cross-linked with glutaraldehyde solution. Another two membranes (PPCAM-PMA and PPCSB-PMA) synthesized by embedding PMA (10% *w*/*w*) were prepared using the same procedure. The PEMs were successfully characterized by FTIR, XRD, SEM, and EDAX studies and assessed for the release of an anti-cancer drug (5-fluorouracil), the (Cu^2+^) removal from aqueous media, and were evaluated for fuel cell application. The PPCAM-PMA and PPCSB-PMA showed superior methanol permeability and proton conductivities compared to the pristine PEMs.

## Data Availability

Not applicable.
